# Association of high titers of anti-carbamylated protein antibodies with decreased bone mineral density in early arthritis patients

**DOI:** 10.1371/journal.pone.0202583

**Published:** 2018-08-17

**Authors:** Cristina Regueiro, Ana M. Ortiz, Maria Dolores Boveda, Santos Castañeda, Isidoro Gonzalez-Alvaro, Antonio Gonzalez

**Affiliations:** 1 Experimental and Observational Rheumatology, Instituto de Investigación Sanitaria - Hospital Clínico Universitario de Santiago, Santiago de Compostela, Spain; 2 Rheumatology Department, Instituto de Investigación del Hospital de La Princesa (IIS-IP), Madrid, Spain; 3 Unit of Diagnosis and Treatment of Congenital Metabolic Diseases, Department of Pediatrics, Instituto de Investigación Sanitaria - Hospital Clínico Universitario de Santiago, Santiago de Compostela, Spain; National Institutes of Health, UNITED STATES

## Abstract

Rheumatoid arthritis (RA) has a negative impact on bone that is partly mediated by anti-citrullinated proteins antibodies (ACPA). These antibodies are associated with erosions, and with juxta-articular and systemic bone loss. Other RA autoantibodies, the anti-carbamylated protein antibodies (anti-CarPA), are independently associated with erosions. However, we do not know if they are also associated with juxta-articular and systemic bone loss. Here, we have addressed this question with data from 548 early arthritis (EA) patients. Bone mineral density (BMD) was assessed by dual-energy x-ray absorptiometry at the lumbar spine (LS), total hip (TH) and metacarpophalangeal joints (MCP). The 25.9% anti-CarPA positive patients did not show significant differences in BMD Z-scores with the negative patients. Nevertheless, this result was due to the similarity between negative and low-positive (below the median of the positive) patients, whereas the high-positive patients showed significant decrease of BMD at LS (β = -0.39, p = 0.01) and TH (β = -0.30, p = 0.02); but not at the juxta-articular bone of MCP. Given the overlap between anti-CarPA and ACPA, we included the two autoantibodies in an analysis that showed significantly lower BMD Z-scores at LS and TH (p< 0.01) only in the ACPA positive/anti-CarPA high-positive subgroup. However, the similar coefficients of regression between the ACPA positive/anti-CarPA high-positive and the ACPA negative/anti-CarPA high-positive subgroups (β = -0.50 *vs*. -0.52 at LS, and β = -0.37 *vs*. -0.30 at TH) suggested an independent association. Overall, these results support a contribution of anti-CarPA to systemic bone loss in EA patients.

## Introduction

Rheumatoid arthritis (RA) is a chronic autoimmune disease characterized by persistent inflammation of the synovial membrane and joint destruction [1]. One of the RA manifestations is bone loss, which can be expressed as erosions, juxta-articular loss or systemic bone loss [2]. The three forms of bone loss differ in their anatomical localization and aspects of their pathogenesis. Erosions are focal losses affecting cortical bone in its full thickness, and the trabecular bone that is immediately underneath [3]. They happen at bone surfaces in contact with inflamed synovia, or tendon sheaths, implicating short-range molecular and cellular inflammatory mediators. Juxta-articular and systemic bone losses are decreases in bone mineral density (BMD), which appear at a distance from the inflamed joints, either near, or far from them, respectively. The separation implies long-range mediators, but inflammation is also a central factor.

Multiple studies show that inflammatory mediators promote osteoclast differentiation and activation, whereas they inhibit osteoblast function [2–5]. Nevertheless, there is an additional mechanism contributing to bone loss in RA, which is mediated by autoantibodies [2,3,6]. A type of autoantibodies, the anti-citrullinated protein antibodies (ACPA) induce bone resorption in *in-vitro* and animal studies. These findings seem to be related to the expression of citrullinated proteins on the surface of osteoclastic precursors, so the attachment of ACPA leads to osteoclast formation and activation, and, consequently, to bone loss [7,8]. In addition, rheumatoid factor (RF) could contribute to bone loss in RA by potentiating the effect of ACPA [9]. Accordingly, multiple epidemiological studies have demonstrated that autoantibodies, mainly ACPA, are associated with the presence and progression of erosions and of juxta-articular bone loss [2,3]. In contrast, the relation of the autoantibodies with systemic bone loss has been less studied, which is unfortunate because systemic bone loss increases the risk of non-traumatic fractures at all localizations [10,11]. Only recently, two studies in cohorts of early RA (ERA) [12] and early arthritis (EA) [13] patients showed that systemic bone loss correlated with the presence of ACPA. In both studies, BMD at the spine and at the hip was lower in ACPA positive than in ACPA negative patients. No association with presence of RF was observed, but in one of the reports, high titer RF potentiated the association of ACPA with low BMD [12]. Two other recent studies have addressed systemic bone loss and autoantibodies, but in patients with long standing RA [14,15]. The two studies reported low BMD at the hip associated with high levels of ACPA.

Other RA autoantibodies, the anti-carbamylated protein antibodies (anti-CarPA), are also associated with the presence, severity and progression of erosions. This association has been observed independently of ACPA, even within ACPA negative patients [16–19]. In addition, the anti-CarPA, which are directed against the post-translational modification of the amino acid lysine to homocitrulline, may be present years before the onset of clinical symptoms, and seem to have a pathogenic role in RA as the ACPA [16,20–22]. However, no study has yet analyzed the possible implication of anti-CarPA on juxta-articular or systemic bone loss, probably, due to their recent discovery and limited availability beyond laboratories performing home-made ELISA.

Therefore, the objective of this work has been to analyze whether the presence of anti-CarPA was associated with juxta-articular or systemic bone loss in a cohort of EA patients. This objective has been pursued by examining the anti-CarPA association with BMD at the MCP joints for juxta-articular bone loss and at the LS and TH for systemic bone loss. The choice of EA patients is justified in the expected gain in sensitivity to detect an effect of the autoantibodies given that bone loss is confounded by other factors in established RA patients [3]. Specifically, the impact of inflammation, treatments (including glucocorticoids) and inactivity are expected to obscure the contribution of antibodies to bone loss at later times. In addition, most EA patients show already their definitive anti-CarPA status [18,20] and bone loss is already frequently present at this time [3].

## Materials and methods

### Patients

A total of 548 patients belonging to the Princesa Early Arthritis Register Longitudinal (PEARL) study were analyzed. This register includes patients submitted to the EA clinic of Hospital Universitario La Princesa (Madrid; Spain) due to suspicion of arthritis of less than a year of evolution. The register protocol includes 5 standardized visits (baseline, 6, 12, 24 and 60 months) at which information about demographics, clinical status, disability, laboratory findings and treatment is collected. Two composite measures of disease activity were used, the Disease Activity Score 28 (DAS28), which assesses the number of tender joints and swollen joints (28 joints maximum), erythrocyte sedimentation rate and global patient health status [23]; and the Hospital Universitario La Princesa Index (HUPI), which includes the same variables but with a modified calculation [24]. At each visit, biological samples are taken, processed and frozen at -80°C. A detailed description of the PEARL study has been previously published [25]. For this work, we used exclusively information and serum samples from the baseline visits of patients recruited from February 2002 to April 2017. All the patients with relevant information and serum were included. However, patients were classified as having RA according to the 2010 ACR/EULAR classification criteria for specific analysis [26]. All patients provided their written informed consent to participate in the study. The PEARL study was approved by the Research Ethics Committee of Hospital Universitario La Princesa (PI-518). This study was approved by the Research Ethics Committee of Galicia (Spain, code 2014/387). Both studies were conducted according to the principles of the Declaration of Helsinki (2013).

### ACPA detection

ACPA were measured using a second-generation anti-citrullinated cyclic peptide enzyme immunoassay (EIA; Euro-Diagnostica Immunoscan RA; positive>50 U/ml) until October 2010, and a third-generation EIA (QUANTA Lite CCP3 IgG and IgA, Inova Diagnostics; positive>40 U/ml) afterwards. Both methods are EIA, but the third-generation assay is able to detect IgA ACPA in addition to IgG antibodies, with no other important differences between these two techniques.

### Determination of anti-CarPA

We used FCS (F-7524, Sigma-Aldrich) as source of proteins for testing anti-CarP reactivity. *In-vitro* carbamylation of proteins from FCS was performed by incubating 4 mg/mL FCS with 1M KCNO, or with 1M KCl as control, during 15 hours at 37°C as previously described [16]. After incubation, the samples were dialyzed against H_2_O with 0.25 NaCl during 24 hours at 4°C. The efficiency and percentage of carbamylation was corroborated by HPLC as previously done [17]. A *Biochrom 30* amino acid analyzer (Biochrom, UK) was used to determine the change of lysine to homocitrulline among the FCS proteins.

IgG anti-CarPA were quantified by ELISA as previously described [16,17]. Separate Nunc MaxiSorp flat-bottom 96 well plates were coated with carbamylated and native FCS overnight at 10 μg/mL in 50 μL of carbonate-bicarbonate buffer 0.1M pH 9.6. The plates were washed with PBS-0.05% Tween and blocked for 6 hours at 4°C with 100 μL of PBS-1% BSA. Diluted serum (50 μL at 1:50 in PBS-1% BSA-0.05%Tween) was incubated on ice overnight. IgG antibodies were detected using ALP-conjugated goat anti-human IgG (Jackson Immunoresearch Europe, UK) and SIGMAFAST p-Nitrophenyl phosphate as a substrate following manufacturer’s recommendations. Reactivity to native FCS was subtracted from the reactivity to carbamylated FCS. Reproducibility of the results was assessed by running all samples in duplicate (mean CV = 4.5% against native FCS and 5.0% against carbamylated FCS) and by including a low titer sample in all plates (mean intraplate CV = 7.4% against native FCS and 4.4% against carbamylated FCS; mean within-plate CV = 6.9% against the two antigens). A standard curve made with serial dilutions from a pool of positive sera was used to measure antibody titers in arbitrary units. The cut-off for positivity was set as the 98% specificity level obtained in the 208 healthy controls. Anti-CarPA levels were classified as negative, low-positive or high-positive, with the two later groups separated by the median of the positives.

### Bone mineral density (BMD) measurements

BMD was assessed by dual-energy X-ray absorptiometry (DXA) on a Hologic QDR-4500 Elite (Bedford, MA, USA) at lumbar spine (LS), hip and hand. Specifically, we analyzed LS-BMD from L2 to L4, total hip (TH), and metacarpophalangeal joints (MCP) from 2^th^ to 5^th^ fingers in the non-dominant hand. BMD was expressed in absolute terms (g/cm^2^ or mg/cm^2^) or as Z-scores. The latter were used to minimize the effect of gender, age and body mass index (BMI). The Z-scores were obtained from the distribution in the respective reference populations for each ethnic group except for MCPs, since the available healthy population data was not enough [27].

### Statistical analysis

The descriptive analysis was performed by calculating the mean and standard deviation (SD) of quantitative variables showing a normal distribution. The median and the interquartile range (IQR) were calculated for those variables not showing a normal distribution. Estimation of the proportions was used to describe qualitative variables. Student’s t test was applied to compare the means of variables with a normal distribution and Mann-Whitney test was used for variables that did not present a normal distribution. The chi-squared test for contingency tables was used to compare the frequencies of qualitative variables. Association between BMD at each anatomic location and anti-CarPA was evaluated with multivariate analysis to account for clinical and demographic factors that differed between the groups of patients. Initial multivariate models included all the variables that were different between the subgroups, as well as, those considered relevant for BMD (age, BMI, smoking, disease activity and cumulative prednisone dose at baseline). The final models were obtained through manual stepwise backward elimination of variables by means of the Bayesian information criterion, removing all variables (except anti-CarPA) with p>0.15. For ascertaining the attributable effect of anti-CarPA, we repeated these multivariate analyses including a variable for ACPA status. All multivariate analyses were performed as generalized linear models with the *glm* command of Stata 12.1 for Windows (Stata Corp LP, College Station, TX). Correction of statistical significance for the number of sites where BMD was determined (n = 3) was applied.

Data availability: all data included in this study is available in the S1 Dataset.

## Results

### Characteristics of the EA patients

**Fig 1:**
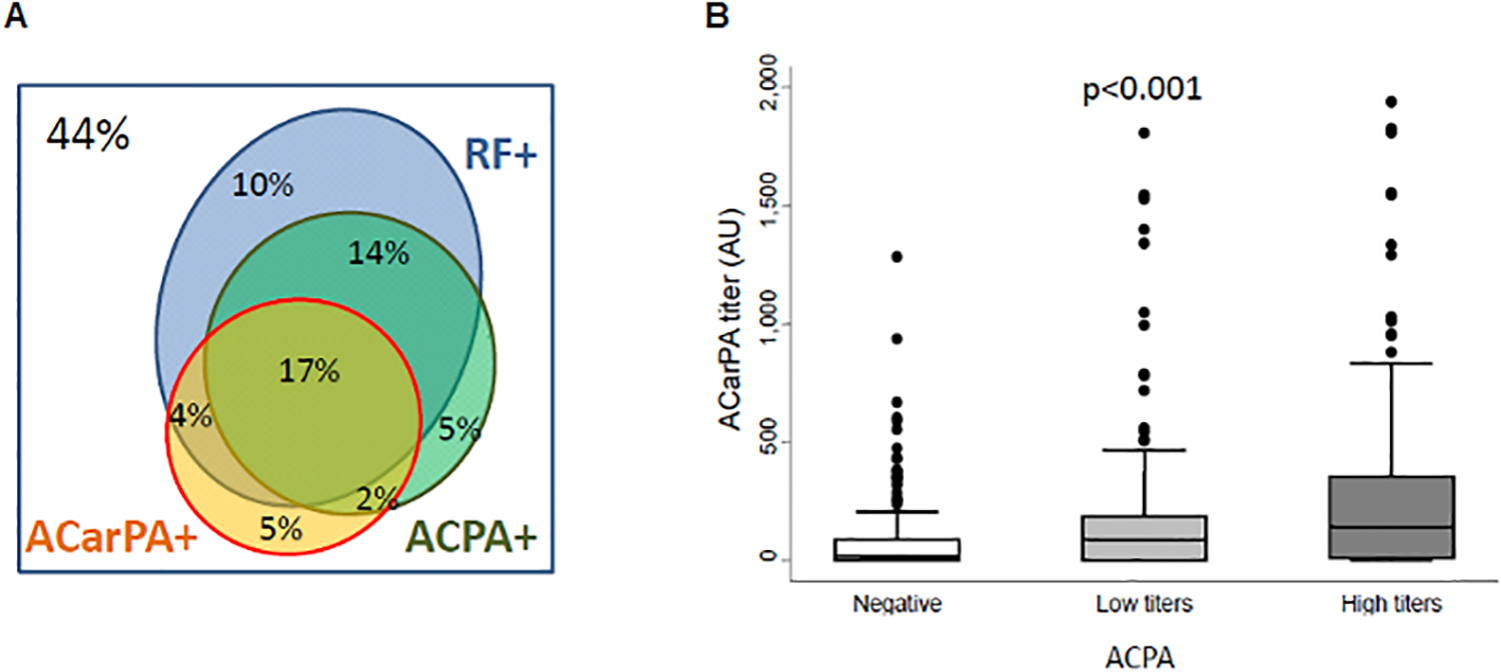
Relationship of anti-CarPA with other RA autoantibodies. A) Proportional Venn diagram showing the overlap between the status of the EA patients for anti-CarPA, ACPA and RF. Data for the three antibodies were available for 532 patients, 44% of which were negative for the three. B) Titers of ACPA and anti-CarPA were correlated.

A fraction of 26% of patients showed anti-CarPA (Fig 1A). These antibodies were predominantly present in patients fulfilling the 2010 RA classification criteria (39.2%), but also detected in the remaining EA patients (11.2%). The anti-CarPA showed a significant overlap with the other RA autoantibodies (Table 1 and Fig 1A). In addition, there was a significant correlation between anti-CarPA and ACPA titers (Fig 1B). The anti-CarPA positive patients were less frequently Caucasian, fulfilled more frequently 2010 RA criteria, and displayed a slightly but significantly higher disease activity, assessed either by DAS28 or HUPI (Table 1). There were also some differences between patients with low and high titers of anti-CarPA, with the latter showing shorter disease duration, and higher disease activity and disability (Table 1).

**Table 1:** Characteristics of the EA patients.

	Anti-CarP negative (n = 406)	Anti-CarP low-positive (n = 68)	Anti-CarP high-positive (n = 74)	P (negative *vs*. positive)	P (low *vs*. high-positive)
Female, n (%)	328 (80.8)	53 (77.94)	62 (83.78)	0.96	0.4
Age, yrs., p50 [IQR]	54.5 [42.0–67.5]	54.8 [46.1–62.7]	54.3 [44.0–64.8]	0.6	0.7
Smoking, n (%)				0.4	0.6
Never	214 (54.6)	34 (52.3)	34 (47.2)		
Ever	94 (24.0)	16 (24.6)	16 (22.2)		
Current	84 (20.7)	15 (23.1)	22 (30.6)		
BMI, p50 [IQR]	26.0 [23.2–29.3]	26.3 [23.5–29.2]	26.2 [22.7–29.3]	0.9	0.6
Menopause (%) No/Yes/NA	61.1/34.5/3.9	54.4/39.7/5.9	58.1/40.5/1.4	0.5	0.3
European, n (%)	353 (87.0)	54 (79.4)	57 (77.0)	0.012	0.7
Prednisone use, n (%)	97 (24.0)	14 (20.6)	20 (27.0)	0.3	0.4
Cumulative prednisone, mg, p50 [IQR]	0 [0–120]	0 [0–83]	0 [0–160]	0.4	0.12
Disease duration, mo, p50 [IQR]	5.0 [2.6–8.2]	6.2 [3.7–9.1]	4.3 [2.7–7.0]	0.8	0.011
2010 RA criteria, n (%)	175 (43.1)	53 (77.9)	60 (81.1)	<0.001	0.6
RF, n (%)	133 (32.8)	51 (75.0)	53 (71.6)	<0.001	0.6
ACPA, n (%)	101 (25.8)	45 (66.2)	53 (73.6)	<0.001	0.3
DAS28, p50 [IQR]	4.0 [3.1–5.1]	3.9 [2.9–5.2]	4.8 [3.5–5.9]	0.045	0.011
HUPI, p50 [IQR]	6.0 [4.0–9.0]	6.5 [4.0–9.0]	8.0 [6.0–10.0]	0.016	0.046
HAQ, p50 [IQR]	0.9 [0.4–1.5]	0.8 [0.3–1.4]	1.0 [0.6–1.6]	0.8	0.022

**Abbreviations**: n: number; IQR: interquartile range; p50: 50^th^ percentile or median; SD: standard deviation; BMI: body mass index; NA: not available; HAQ: Health Assessment Questionnaire.

### Association of low BMD at LS and TH with anti-CarPA

Analysis of the relationship between BMD and anti-CarPA for the three assessed anatomical locations was performed in several ways (Tables 2 and 3). When the anti-CarPA were considered as either positive or negative (Table 2), the positive EA patients showed significantly lower BMD in absolute terms (mg/cm^2^) than the negative patients at TH (p = 0.02). This association did not persist after correcting by the number of sites where BMD was assessed (p = 0.06). No other comparison between anti-CarPA positive and negative patients showed significant differences. However, stratification of the patients in three levels (negative, low-positive and high-positive for anti-CarPA) showed that these results were attributable to the lack of differences between low-positive and negative patients (Table 3). In contrast, the high-positive patients showed a significantly lower BMD Z-scores than the negative patients both at LS and at TH (p = 0.01 and p = 0.02, respectively), although only the first of these associations persisted after correcting for the number of BMD sites (p = 0.03 and 0.06, respectively). No such differences were demonstrated at the MCP joints.

**Table 2:** Association of BMD at different locations with anti-CarPA.

Location	BMD measure	Coefficient ^a^	SE	P
LS	mg/cm^2^	-0.02	0.01	0.16
	Z-score	-0.18	0.12	0.14
TH	mg/cm^2^	-0.03	0.01	0.02
	Z-score	-0.15	0.10	0.12
MCP ^b^	mg/cm^2^	0.00	0.01	0.59

BMD was measured either as mg/cm^2^ or as Z-scores, and anti-CarPA were considered as positive/negative.

^a^ Multiple linear regression analysis including as covariates sex, body mass index (BMI), age at initiation, and menopause status

^b^ Z-scores for BMD at MCP joints are not available

Abbreviations: SE: standard error of the coefficient.

**Table 3:** Association of BMD at different locations with anti-CarPA stratified as negative, low-positive and high-positive.

Location	BMD measure	anti-CarPA	Coefficient ^a^	SE	P
LS	mg/cm^2^	negative ^b^	ref.		
		low-positive	0.01	0.02	0.7
		high-positive	-0.04	0.02	0.03
	Z-score	negative ^a^	ref.		
		low-positive	0.05	0.16	0.8
		high-positive	-0.39	0.16	0.01
TH	mg/cm^2^	negative ^c^	ref.		
		low-positive	-0.03	0.02	0.10
		high-positive	-0.03	0.02	0.08
	Z-score	negative ^c^	ref.		
		low-positive	0.00	0.13	1.0
		high-positive	-0.30	0.13	0.02
MCP ^d^	mg/cm^2^	negative ^a^	ref.		
		low-positive	0.01	0.01	0.4
		high-positive	0.00	0.01	0.4

BMD was measured either as mg/cm^2^ or as Z-scores.

^a^ Multiple linear regression analysis including as covariates sex, BMI, age at initiation, and menopause status

^b^ Idem as ^a^ plus a covariate for ethnicity

^c^ Idem as ^a^ plus a covariate for disease activity measured with HUPI

^d^ Z-scores for BMD at MCP joints are not available

The multivariate analysis on the previous paragraph was done with adjustment for variables that showed significant association with BMD. The variables included at all locations were sex, age, BMI and menopause status in women. In addition, ethnicity (European/non-European), and disease activity were significantly associated in specific analyses and, were incorporated in the corresponding models (Table 3). Other clinical and demographic variables, comprising fulfillment of the 2010 RA criteria and disease duration, did not show significant association with BMD.

### Relationship between ACPA and anti-CarPA in the association with BMD

Given the significant overlap and correlation between anti-CarPA and ACPA in our cohort (Fig 1A and 1B), it was uncertain to which antibody the association could be attributed. In consequence, we included the two autoantibodies in multivariate analysis trying to clarify their relative contributions. The results suggested that high titer anti-CarPA were independently associated with BMD at LS and at TH (Table 4). In more detail, the association with BMD expressed as Z-scores was only significant in the ACPA positive/anti-CarPA high-positive subgroup of patients both at LS and at TH (p = 0.007 and 0.005, respectively). These results persisted after correction by the number of BMD sites (p = 0.02 and 0.015, respectively). However, the coefficients of regression were similar between the ACPA positive/anti-CarPA high-positive subgroup and the ACPA negative/anti-CarPA high-positive subgroup (β = -0.50 *vs*. -0.52 at LS, and β = -0.37 *vs*. -0.30 at TH). These similarities suggested independence from the presence of ACPA. Nevertheless, BMD on the ACPA negative/anti-CarPA high-positive subgroup was not significantly different from the reference seronegative patients likely due to the relative small number of subjects in this stratum (Table 4).

**Table 4:** Conditional nalysis of the association of BMD with anti-CarPA and ACPA.

Location	anti-CCP	anti-CarPA (n)^a^	Coefficient ^b^	SE	p
LS	negative	negative (291)	ref.		
		low-positive (23)	-0.17	0.27	0.5
		high-positive (19)	-0.52	0.30	0.09
	positive	negative (101)	-0.25	0.14	0.09
		low-positive (45)	0.06	0.20	0.8
		high-positive (53)	-0.50	0.19	0.007
TH	negative	negative	ref.		
		low-positive	-0.27	0.19	0.16
		high-positive	-0.30	0.22	0.17
	positive	negative	-0.10	0.10	0.3
		low-positive	0.10	0.14	0.5
		high-positive	-0.37	0.13	0.005

Only the strongest associations from Table 3 were considered here.

^a^ Number of patients in each stratum, which are identical for LS and for TH (not shown).

^b^ Multiple linear regression analysis including as covariates sex, BMI, age at initiation, and menopause status for LS, and the same covariates plus a covariate for disease activity measured with HUPI for TH.

## Discussion

In this study, we have found significant lower BMD at LS and TH of patients with high titers of anti-CarPA, suggesting that anti-CarPA at high titers could contribute to systemic bone loss in EA patients in an independent way. By contrast, BMD at the MCP joints, which reflect juxta-articular bone loss, was not associated with anti-CarPA. To the best of our knowledge, this is the first study analyzing the relationship between the presence of anti-CarP antibodies and systemic or juxta-articular bone loss.

The coexistence of different autoantibodies in patients with RA hampers the determination of their relative contribution to the complications that may appear throughout the disease evolution. This task has been further complicated in the current study by the association of systemic bone loss only in the patients with high titers of anti-CarPA. This circumstance increased the division of the patients into a larger number of strata. Even with these limitations, the evidence of an independent contribution of high anti-CarPA when accounting for ACPA was consistent in the different performed analyses. They showed that the BMD decrease was similar in ACPA positive and negative patients when they presented high titers of anti-CarPA. In addition, RF did not contribute significantly to that association, as has already been reported in a previous analysis [13].

The finding of a dose-effect on the association of anti-CarPA with systemic bone loss is reminiscent of some results obtained with ACPA. In effect, a clear dose-effect of ACPA titers was reported in the association with decreased BMD at the hip in ERA patients [12] and in established RA patients [14,15]. Also, a dose-effect was reported at LS in the single study on established RA patients that analyzed this anatomical location [14]. However, not all BMD results are concordant. We did not observe a dose-response association of decreased BMD with ACPA at either LS or TH (not shown), and the same happened in the study assessing BMD at LS in ERA patients [12]. More concordance has been observed in the analyses of the relationship between ACPA and erosions. A dose-effect on the association has been reported in most studies that have addressed this relationship [28–30]. However, no specific account of the possible mechanism has been proposed. It could be simply a matter of sensitivity: with bone loss requiring a threshold antibody level. This possibility is suggested by the dose-effect relationship described for osteoclast activation [8]. Alternatively, the dose-effect of ACPA could be related with the diversity of the recognized antigens, as subjects with high titers show a wider range of specificities [31]. At present, we can only speculate that something similar could happen regarding the dose-effect of anti-CarPA on BMD at LS and TH.

The association of ACPA with bone loss was known for more than a decade before their capacity of activating osteoclasts was discovered [7]. The mechanistic studies implicate only a fraction of ACPA, at least those against citrullinated vimentin and citrullinated enolase [7,8]. Other ACPA, as those against citrullinated fibrinogen, seem to be unable to activate osteoclasts [8]. It is assumed that this activation explains ACPA association with bone loss in its three presentations: erosions, juxta-articular loss and systemic bone loss [2]. The difference in pathogenesis between them depends on other contributing factors. Some notable ones are the contact with inflamed synovial tissue and the presence of local cytokines including RANKL for local bone loss, whereas other cytokines as DKK1 contribute to systemic bone loss [3,32–34]. By analogy, it could be expected that the mechanism linking anti-CarPA with bone loss will be shared between its three aspects. Also, it is possible that not all anti-CarPA are able to induce bone loss. However, we still do not know the natural antigens recognized by anti-CarPA and we will need to discover them before exploring this question. Fortunately, there are already two natural antigens, albumin and alpha-1 antitrypsin, that have some experimental support as anti-CarPA targets [35,36].

The absence of association of BMD loss at the MCP joints with anti-CarPA found here should be interpreted in the light of the results obtained for ACPA. ACPA was not associated with BMD loss assessed with DXA at the MCP in a previous study [13], whereas they were associated in other studies [37–39]. Some of these latter studies were also done in EA patients [38], or in ACPA positive subjects with arthralgia [39], in whom low level of bone loss due to incipient pathology is expected. However, these studies used high resolution quantitative computed tomography [37,39] or digital X-ray radiogrammetry [38], both of which are more sensitive technologies than DXA at the MCP joints [40,41]. Therefore, it is likely that DXA limited sensitivity could explain our negative results. Consequently, the anti-CarPA role on juxta-articular bone loss remains uncertain, awaiting studies conducted with other technologies.

The implications of our results are threefold. They indicate that anti-CarPA at high titers contribute to systemic bone loss. This function extends the involvement of anti-CarPA in the bone changes observed in RA beyond their established role in erosions [16–19]. In addition, the results suggest a parallelism with ACPA in the stimulation of osteoclastogenesis and remark the need of studies addressing this point. Of considerable interest in this respect will be the clarification of the relative contribution of the two autoantibodies and the possibility of interactions suggested by our analysis. In addition, our results reinforce the possibility that anti-CarPA could be useful biomarkers of disease severity. They have been associated with increased radiographic progression, disease activity, disability and mortality in several studies [16–19,42–44]. The association with systemic bone loss reported here adds to the list of characteristics that will deserve a more aggressive treatment of the patients bearing anti-CarPA.

In conclusion, we have found decreased BMD at LS and TH in EA patients with high titers of anti-CarPA. This decrease is evidence of a reduction in systemic bone mass that was, at least in part, independent of the presence of ACPA. Nevertheless, more studies are needed to confirm our results and establish the mechanism and the clinical significance of these findings.

## Supporting information

S1 Dataset Raw data with all the variables and patients considered in this study. (XLS)
